# Supplementary material for: AI-assisted selection of mating pairs through simulation-based optimized progeny allocation strategies in plant breeding
Source: Front Plant Sci. 2024 Mar 28;15:1361894. doi: 10.3389/fpls.2024.1361894 (PMC11138345; doi:10.3389/fpls.2024.1361894)
Supplement: Supplementary file 1 [file DataSheet_1.zip › Figure S1 - S3, Table S1 - S2.PDF]

frontiers

Supplementary Material for

"AI-assisted selection of mating pairs through simulation-based optimized progeny allocation strategies in plant breeding"

1 Supplementary Figures and Tables

1.1 Supplementary Figures

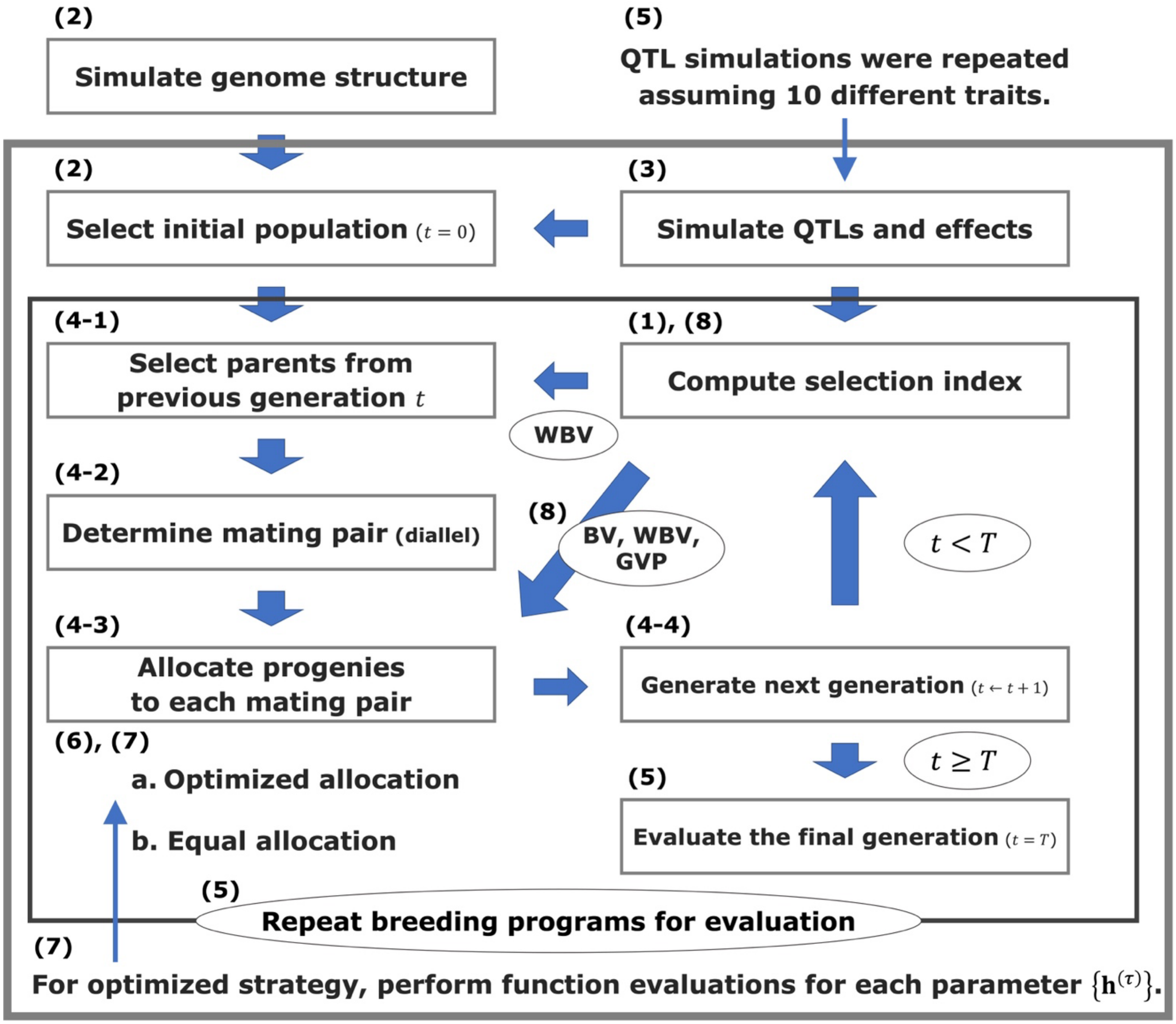

Supplementary Figure 1. Flowcharts for the overview of our simulation study. The numbers in parentheses indicate the order in which they will be explained.

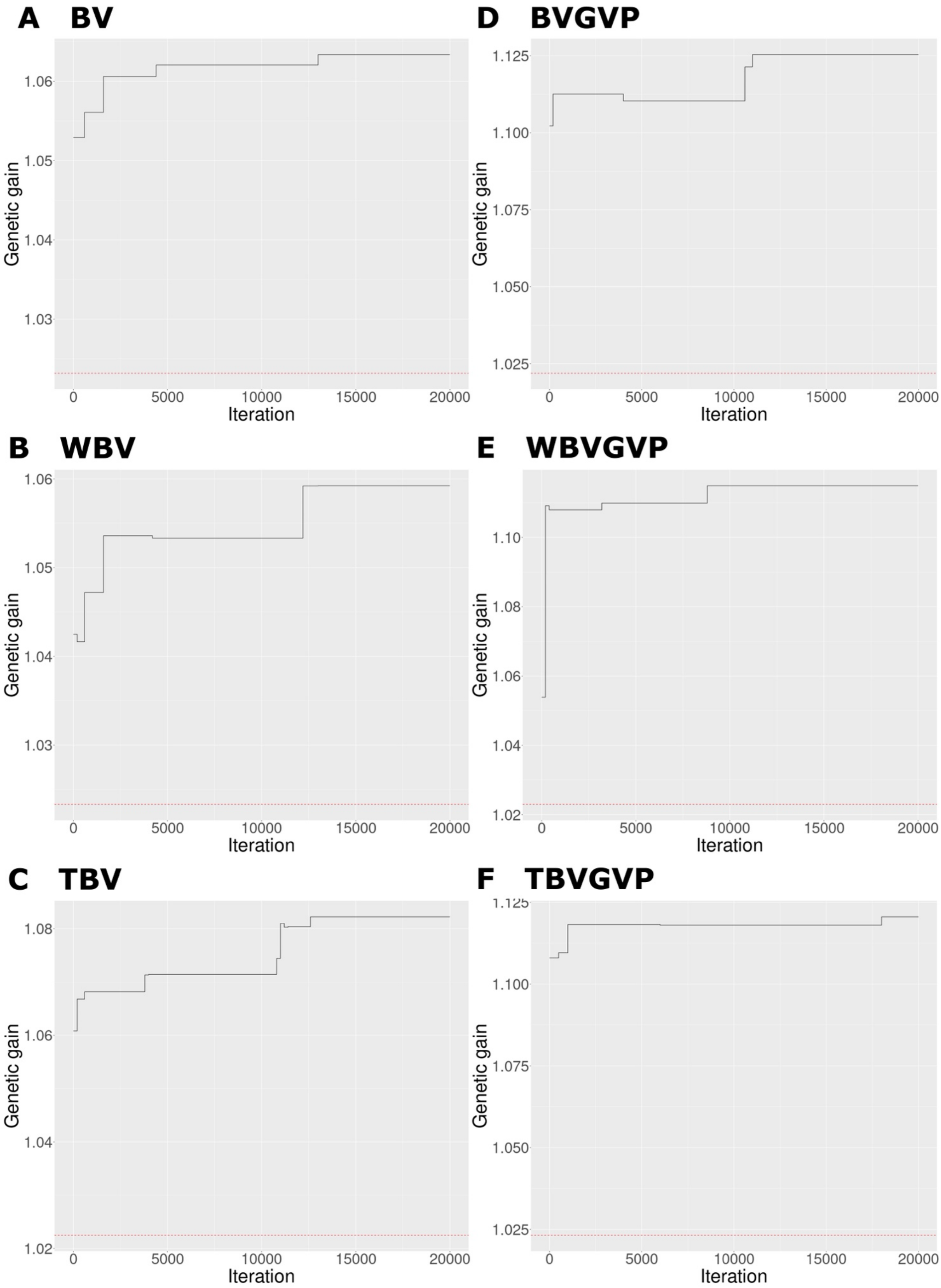
A BV
1.06
1.05
1.04
1.03
Genetic gain
0
5000
10000
15000
20000
Iteration
D BVGVP
1.125
1.100
1.075
1.050
1.025
Genetic gain
0
5000
10000
15000
20000
Iteration
B WBV
1.06
1.05
1.04
1.03
Genetic gain
0
5000
10000
15000
20000
Iteration
E WBVGVP
1.10
1.08
1.06
1.04
1.02
Genetic gain
0
5000
10000
15000
20000
Iteration
C TBV
1.08
1.06
1.04
1.02
Genetic gain
0
5000
10000
15000
20000
Iteration
F TBVGVP
1.125
1.100
1.075
1.050
1.025
Genetic gain
0
5000
10000
15000
20000
Iteration

Supplementary Figure 2. Change in function values for each allocation strategy optimized by the StoSOO algorithm. The black solid line shows the change in function values (expected final genetic gains evaluated by 10,000 simulations) for each allocation strategy optimized by the StoSOO algorithm. The red dashed line shows the expected final genetic gain based on the equal allocation strategy. Each subplot corresponds to the result for each optimized strategy as follows; (**A**) BV, (**B**) WBV, (**C**) TBV, (**D**) BVGVP, (**E**) WBVGVP, (**F**) TBVGVP. The abbreviation of each strategy is as same as that in Figure 2.

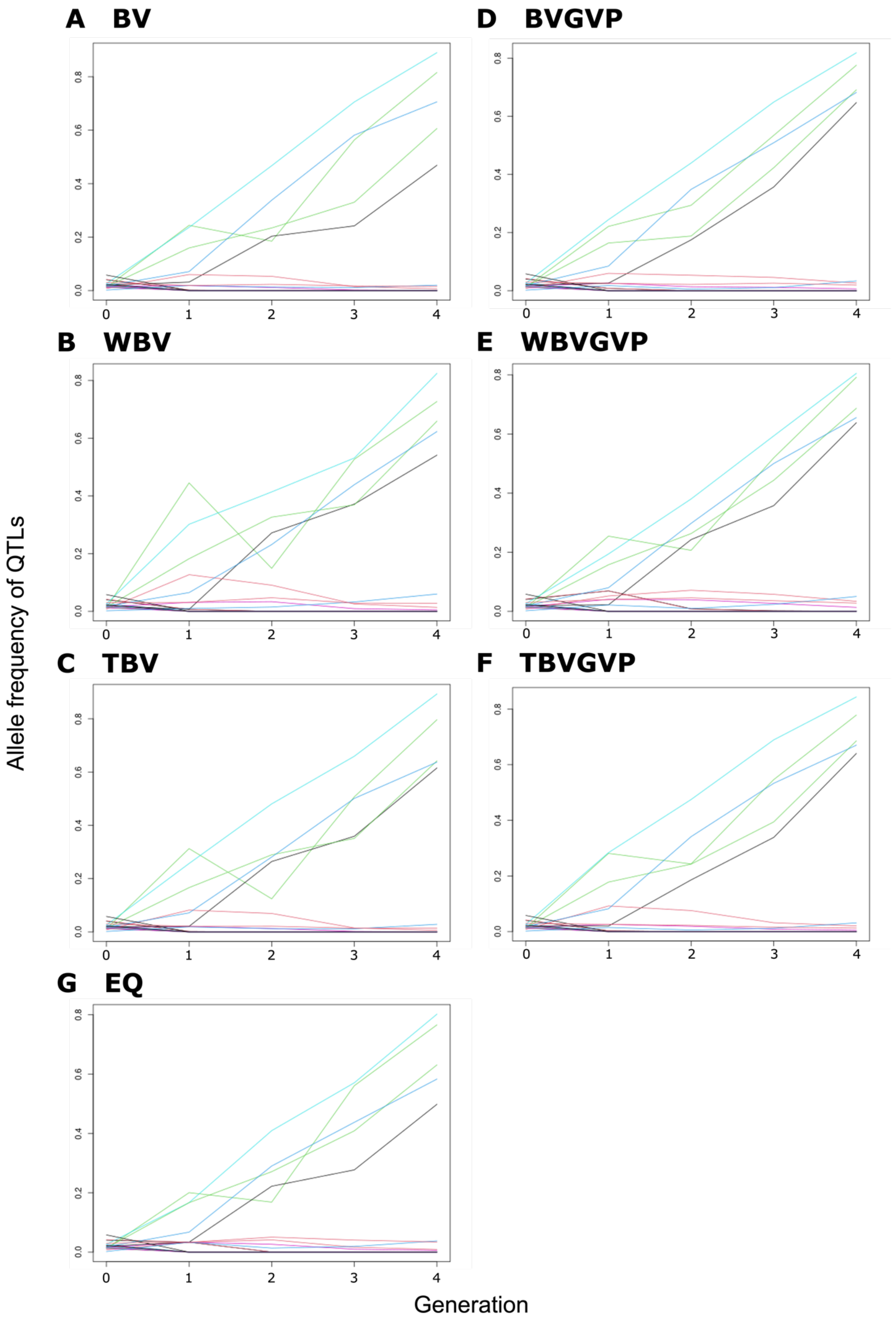
A BV
B WBV
C TBV
D BVGVP
E WBVGVP
F TBVGVP
G EQ
0.0
0.2
0.4
0.6
0.8
0
1
2
3
4
Allele frequency of QTLs
Generation

Supplementary Figure 3. Change in allele frequencies of 20 QTLs for each allocation strategy, including equal allocation. Each color corresponds to the change in allele frequency of different QTL (expected allele frequencies evaluated by 1,000 simulations). Each subplot corresponds to the result for each optimized strategy as follows; (**A**) BV, (**B**) WBV, (**C**) TBV, (**D**) BVGVP, (**E**) WBVGVP, (**F**) TBVGVP, (**G**) EQ. The abbreviation of each strategy is as same as that in Figure 2.

1.2 Supplementary Tables

Supplementary Table 1. Optimized weighting parameters $\mathbf{h}^{(\tau)}$ in different generations $\tau = 0, \dots, T-1$ for WBV and WBVGVP strategies.

	**WBV**		**WBVGVP**	
**Generation ($\boldsymbol{\tau}$)**	WBV	GVP	WBV	GVP
**0**	1.22	–	0.33	0.33
**1**	0.85	–	1.00	1.00
**2**	1.88	–	1.67	1.67
**3**	1.67	–	1.00	1.00

Supplementary Table 2. Optimized weighting parameters $\mathbf{h}^{(\tau)}$ in different generations $\tau = 0, \dots, T-1$ for WBV and TBVGVP strategies.

	**TBV**			**TBVGVP**		
**Generation ($\boldsymbol{\tau}$)**	BV	WBV	GVP	BV	WBV	GVP
**0**	1.00	0.33	–	1.00	0.33	0.33
**1**	0.33	1.00	–	1.00	0.33	1.00
**2**	1.89	1.00	–	1.67	1.00	1.00
**3**	1.67	1.67	–	1.00	1.00	1.00
